# Letter to the Editor: Global burden, trends and forecast analysis of extremity fractures based on GBD 2021

**DOI:** 10.1097/JS9.0000000000002678

**Published:** 2025-06-12

**Authors:** Xianwen Sun, Jun Fei

**Affiliations:** a Department of Orthopedics, Zhejiang Chinese Medical University Affiliated Hospital of Integrated Traditional Chinese and Western Medicine, Hangzhou, Zhejiang, China; b The Second School of Clinical Medicine, Zhejiang Chinese Medical University, Hangzhou, Zhejiang, China

*Dear Editor,*

We read the analysis by Wang *et al* with great interest. The authors distil three decades of Global Burden of Disease (GBD) data into a narrative for seven categories of extremity fractures (EFs) and argue that their forecasts should guide orthopedic planning worldwide. In line with the TITAN 2025 guideline on AI transparency^[1]^, a generative AI-based language model was used solely for language polishing and had no bearing on the scientific content.

First, diagnostic heterogeneity challenges any global estimate. The study merges ICD‑10 codes S40–S99 into seven anatomic buckets, but low socio‑demographic index (SDI) regions often code limb injuries inconsistently or omit imaging altogether; under‑ascertainment in sub‑Saharan datasets may reach 40 percent^[2]^. A sensitivity check against WHO sentinel registries could reassure readers that the observed gradients are not artifacts of missing data.

Second, the choice of estimated annual percentage change (EAPC) combined with a naive ARIMA model may over‑smooth real inflection points. Hip‑fracture incidence in several high‑income nations plateaued after 2010 as prophylactic therapy spread. Join‑point or Bayesian segmented regression would capture such bends, and forecast intervals should be shown. A network meta‑analysis of 68 randomized trials indicates pharmacological prophylaxis can alter fracture trajectories within 5 years^[3]^; modeling such covariates could refine projections.

Third, reporting only age‑standardized rates risks masking the absolute service burden. A stable ASR may hide a growing raw case count in ageing populations. Presenting absolute numbers for 2030 and 2040, plus apportioning burden to modifiable risks, would help prioritize action. For example, a multicenter trial found changing imaging strategy doubled the recorded rate of pediatric forearm “cracks” of dubious clinical relevance^[4]^ – practice shifts that ripple through projections.

Fourth, the clinical and economic implications deserve deeper exploration. Hip and femoral‑shaft fractures drive mortality and resource use, whereas phalangeal breaks rarely do. A cost‑per‑DALY model could reveal which fracture groups offer the best prevention dividend. Meta‑analysis across cohort studies links osteoporosis therapy to lower all‑cause mortality^[5]^; coupling this evidence to forecast volumes might guide ministries in allocating limited funds.

In sum, Wang *et al* lay an important foundation, yet future iterations would benefit from three targeted enhancements: first, multi-source calibration to reduce estimation bias in low-SDI settings; second, non-linear join-point forecasting to capture genuine inflection points more accurately than a single EAPC slope; and third, coupling DALY projections with cost-per-DALY analyses to help policy-makers prioritize prevention spending – together these refinements will better equip clinicians and decision-makers to navigate the coming fracture tide.These suggestions aim to enhance the scientific rigor and clinical interpretability of the article by Wang et al.^[6]^, which provides valuable epidemiological insights based on GBD 2021 data and deserves attention from both orthopedic researchers and global health policymakers.

## Data Availability

No new data were generated or analyzed.

## AI assistance

A generative AI-based language model was employed solely for grammar and style editing.
